# Unstable angina early after aortic valve replacement surgery in a female patient with normal coronary arteries preoperatively – a case report

**DOI:** 10.1186/1749-8090-4-29

**Published:** 2009-07-02

**Authors:** Sybille Gruber, Choi-Keung Ng, Christian Schwarz, Johann Auer

**Affiliations:** 1 Department of Cardiology and Intensive Care, General Hospital Braunau, Austria; 2 Department of Cardiac Surgery, General Hospital Wels, Austria; 3 Department of Cardiology and Intensive Care, General Hospital Simbach, Germany

## Abstract

**Background:**

Angina pectoris early after aortic valve replacement surgery in patients with previously normal coronary arteries may be life threatening and has to be assessed immediately.

**Case report:**

12 weeks after aortic valve replacement surgery, a 60-year-old female patient was referred for evaluation of recent onset of severe chest pain on mild exertion and at rest. Coronary angiography showed severe stenosis nvolving the left coronary ostium and the left main stem. The patient was urgently referred for bypass surgery and had an uneventful postoperative recovery.

**Conclusion:**

A high degree of suspicion is needed for early recognition and aggressive management of this rare but serious complication.

## Background

Unstable angina is rare, but may be life-threatening in patients in the early postoperative period following aortic valve replacement with normal preoperative coronary arteries.

## Methods and results

We report the case of a 60-year-old female patient undergoing valve replacement surgery for symptomatic aortic valve stenosis. Preoperative echocardiographic assessment revealed a severely calcified aortic valve with a calculated aortic valve area of 0.8 ccm. Mean pressure gradient was 55 mmHg and left ventricular ejection fraction was well preserved.

The patient was free of angina and reported dyspnoea on exertion.

Preoperative coronary angiography revealed normal coronary arteries.

Valve replacement surgery was performed using a Sorin 23 mm mechanical valve prosthesis. Early postoperative recovery was unremarkable.

12 weeks after surgery the patient was referred for evaluation of recent onset of severe chest pain on mild exertion and at rest.

ECG revealed severe ST-segment depression in leads V2-5 during episodes of chest pain.

Coronary angiography showed a 90% diameter reduction involving the left coronary ostium and the left main stem.

The patient was urgently referred for bypass surgery and had an uneventful postoperative recovery.

## Conclusion

Angina pectoris early after aortic valve replacement surgery in patients with previously normal coronary arteries may be life threatening and has to be assessed immediately. A high degree of suspicion is needed for early recognition and aggressive management of this rare but serious complication.

## Introduction

Unstable angina early after aortic valve replacement in patients with normal coronary arteries in the preoperative angiography is rare.

Generally, possible differential diagnoses of postoperative angina pectoris in patients undergoing mechanical aortic valve replacement are coronary embolism, progression of coronary heart disease in patients with coronary atherosclerosis, graft occlusion in patients with concomitant aortocoronary bypass (ACBP) and iatrogenic coronary ostial/main stem stenosis.

Many cases of iatrogenic coronary ostial/main stem stenoses have been reported since the late 1960ies, all cases showing similar patterns – sudden onset of angina pectoris 3–6 months postoperatively in patients without or with only mild coronary artery disease – and similar histologic findings – intimal fibrous proliferation of one or both coronary ostia [1-9].

## Patient and methods

We report the case of a 60-year-old moderately obese female patient, who was referred for evaluation of recent onset dyspnea on exertion. Physical examination revealed a 3/6 crescendeo-decrescendo systolic murmur at the aortic valve.

**Figure 1:**
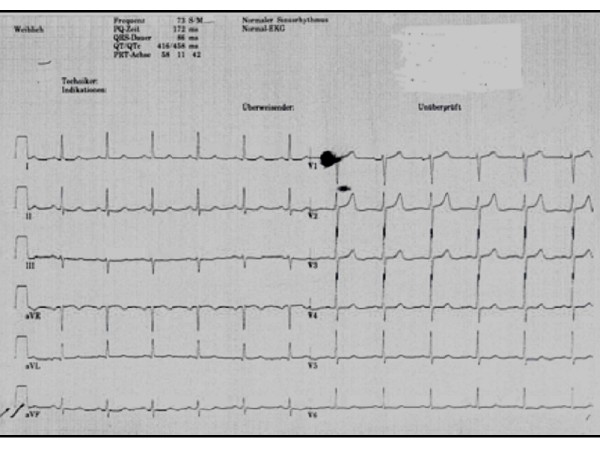
**Preopoerative ECG: Regular sinus rhythm**.

Electrocardiography showed regular sinus rhythm without additional findings (Fig. 1)

Echocardiographic assessment revealed a severely calcified aortic valve with a calculated valve area of 0,80 cm^2 ^and a mean pressure gradient of 55 mmHg.

Left ventricular ejection fraction was well preserved with left ventricular wall thickness of 14 mm (septal and posterolateral).

**Figure 2:**
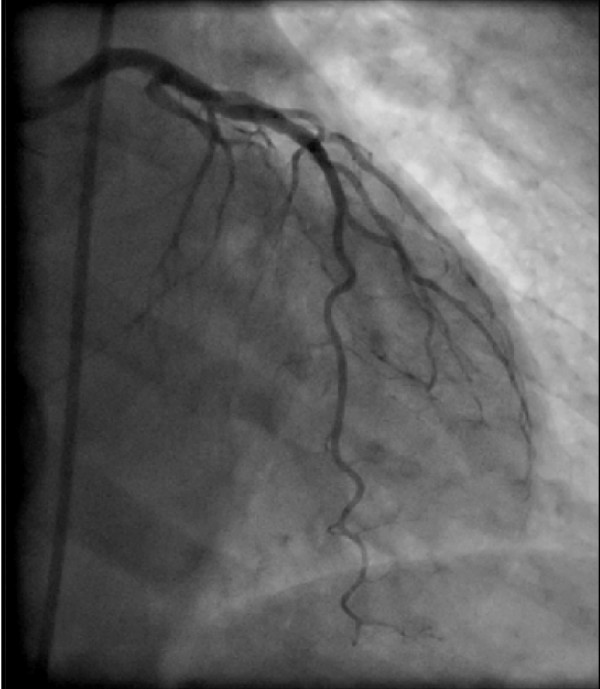
**Preoperative coronary angiography (RAO view) showing the left coronary artery**.

Preoperative coronary angiography revealed completely normal coronary arteries (Fig 2).

The patient underwent aortic valve replacement surgery. The procedure was performed using mini-sternotomy with a single 2-stage venous cannula and normothermic cardiopulmonary bypass. The use of normothermic techniques has been reported to confer several advantages over conventional hypothermia, such as reduced bleeding and requirements for electrical defibrillation, shorter intubation times, and improved hemodynamic parameters postoperatively. Myocardial protection with cold antegrade and retrograde St. Thomas' cardioplegic (II) solution was obtained immediately after aortic cross clamping. The aortic valve is exposed through an oblique aortotomy incision made well above the orifice of the right coronary artery. The severely calcified stenotic aortic valve was excised, replaced with a Sorin 23 mm mechanical valve prosthesis, attached with subannular mattress sutures of 2-0 Ethibond (Ethicon, Sommerville, New Jersey, USA).

Early postoperative recovery was unremarkable.

**Figure 3:**
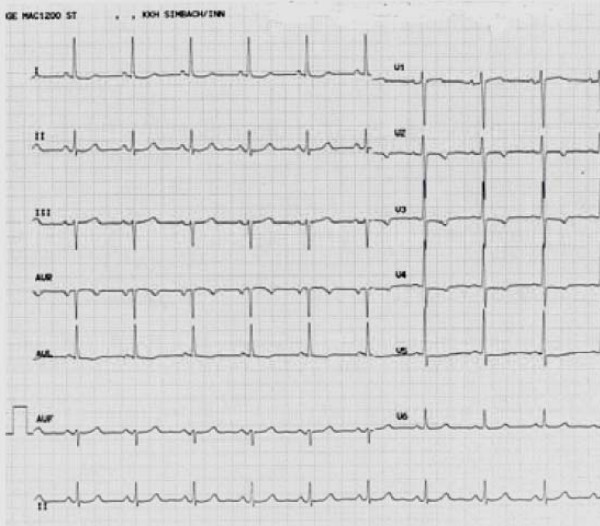
**ECG at admission three months after valve replacement with ST-T-abnormalities in the anterior leads**.

12 weeks after surgery the patient was referred for evaluation of recent onset of severe chest pain on mild exertion and at rest. ECG at admission showed inverted T-waves in leads V2 to V5 and in lead aVL (Fig. 3).

**Figure 4:**
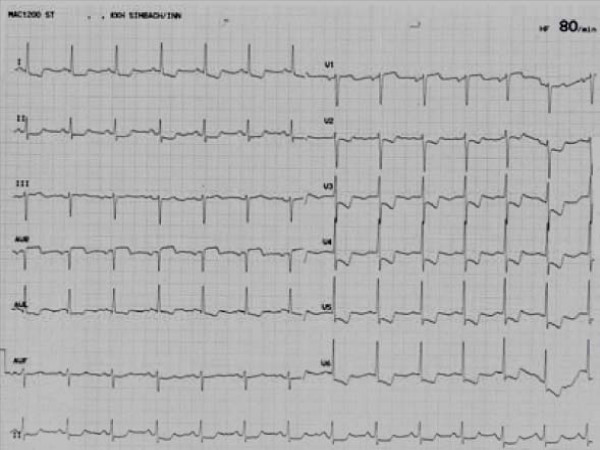
**ST segment depressions during severe angina at rest**.

During episodes of chest pain ECG revealed severe ST-segment depression in leads V2 to V5 (Fig 4).

**Figure 5:**
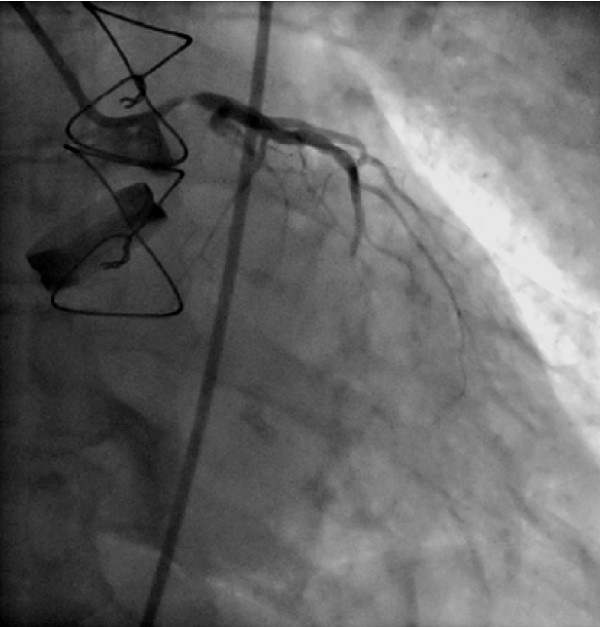
**Postoperative coronary angiography (RAO view) showing the left coronary artery**.

Coronary angiography showed a 90% diameter reduction involving the left coronary ostium and the left main stem (Fig 5).

The patient was urgently referred for bypass surgery with a left internal mammary artery graft to the LAD and a left radial artery graft to the circumflex artery.

Postoperative recovery was unremarkable. Four months later she is doing well without chest pain or signs of myocardial ischemia.

## Discussion

The present case underlines the importance of early diagnosis and treatment if angina pectoris occurs after aortic valve replacement.

Coronary ostial stenoses can be detected in 0,1% of coronary angiographies in unselected patients [10,11]. Apart from atherosclerosis as the prime genesis, thromboses as well as infections (lues) can provoke ostial stenoses. There has also been a report about solitary ostial stenosis in a patient with Takayasu's arteritis [12].

Iatrogenic coronary ostial stenosis is a well recognized, but uncommon and potentially life threatening complication of aortic valve replacement. Symptoms include chest pain during exercise or at rest, sudden onset of acute heart failure and acute pulmonary edema. Usually, symptoms occur within the first 6 months, though they may occur up to 30 months after the operation [1].

Main stem stenosis after aortic valve replacement was first recognised in 1969 by Trimble et al [2]. who described the cases of three patients who underwent surgery for aortic valve stenosis and/or insufficiency in 1965. Three to four months after the operation they developed angina pectoris, in each case coronary angiography showed severe stenosis of either one or both coronary ostia. According to Lesage et al. the incidence of this severe complication may be as high as 0.9% [3] – however, incidence is decreasing, because of improved operative techniques [4].

Several pathogenic mechanisms have been suggested: aortic root fibrosis secondary to turbulent flow around the prosthesis [13]; the presence of perfusion catheters during valve surgery that produce local pressure necrosis and subsequent intimal proliferation leading to obstruction of the coronary ostia [2]; balloon inflation in the proximal parts of the vessels; turbulence that causes coronary artery intimal injury that might explain the lesions often found distant to the adherence of the cannulation devices; immunologic reaction after valve replacement with heterograft [5].

There may also be a genetic predisposition for developing this complication since 70% of the affected individuals as compared to 10–15% in a control group had an epsilon 4 allele apolipoprotein E genotype [6].

Instrumentation with minimal trauma of the left main stem is most likely the cause of early postoperative stenosis after aortic valve replacement surgery [7,8,14].

Avoiding cannulation of the coronary ostia for antegrade cardioplegia, but instead using retrograde delivery as an alternative method for myocardial perfusion during open heart surgery may reduce the risk of postoperative coronary ostial or left main stem stenosis [9,15].

## Conclusion

If recurrent or newly onset angina pectoris occurs early after aortic valve

Replacement, a high degree of suspicion os warranted to recognize this rare but life threatening complication of coronary ostial stenosis, even if preoperative coronary angiography did not show coronary heart disease.

New operative techniques that reduce manipulation and consequently avoid trauma of the coronary vessels may prevent postoperative coronary ostial stenosis. Future studies in patients undergoing this procedure using retrograde cardioplegia only will have to prove this hypothesis.

## Consent

Written informed consent was obtained from the patient for publication of this case report and accompanying images. A copy of the written consent is available for review by the Editor-in-Chief of this journal.

## Competing interests

The authors declare that they have no competing interests.

## Authors' contributions

SG was the main author and wrote the article. CKN was the surgical consultant, was involved in data collection and revised the manuscript. CS was the surgical consultant was involved in data collection and interpretation. JA was the cardiology consultant and gave final approval of the manuscript. All authors have read and approved the final manuscript.
